# SARS-CoV-2 spike protein antibody titers after the fourth dose of BNT162b2 vaccine among Japanese patients undergoing hemodialysis: a single-center study

**DOI:** 10.3389/fimmu.2024.1412918

**Published:** 2024-08-21

**Authors:** Shun Watanabe, Toyoaki Sawano, Hiroaki Saito, Akihiko Ozaki, Masatoshi Wakui, Tianchen Zhao, Chika Yamamoto, Yurie Kobashi, Takeshi Kawamura, Akira Sugiyama, Aya Nakayama, Yudai Kaneko, Hiroaki Shimmura, Masaharu Tsubokura

**Affiliations:** ^1^ Department of Urology, Jyoban Hospital of Tokiwa Foundation, Iwaki, Fukushima, Japan; ^2^ Department of Surgery, Jyoban Hospital of Tokiwa Foundation, Iwaki, Fukushima, Japan; ^3^ Department of Radiation Health Management, Fukushima Medical University School of Medicine, Fukushima, Fukushima, Japan; ^4^ Department of Internal Medicine, Soma Central Hospital, Soma, Fukushima, Japan; ^5^ Breast and Thyroid Center, Jyoban Hospital of Tokiwa Foundation, Iwaki, Fukushima, Japan; ^6^ Department of Laboratory Medicine, Keio University School of Medicine, Tokyo, Japan; ^7^ Department of Internal Medicine, Seireikai Group Hirata Central Hospital, Fukushima, Japan; ^8^ Isotope Science Center, The University of Tokyo, Tokyo, Japan; ^9^ Laboratory for Systems Biology and Medicine, Research Center for Advanced Science and Technology, The University of Tokyo, Tokyo, Japan; ^10^ Medical and Biological Laboratories Co., Ltd, Tokyo, Japan

**Keywords:** SARS-CoV-2, hemodialysis, chronic kidney failure, fourth dose of vaccine, IgG antibody titers, BNT162b2 vaccine

## Abstract

Patients undergoing hemodialysis are particularly vulnerable to severe outcomes of SARS-CoV-2 infection, with mortality rates higher than that of the general population. Vaccination reduces the risk of adverse outcomes, with booster doses being particularly beneficial. However, limited data are available on the effectiveness of subsequent vaccinations or their effect on increasing antibody levels. This single-center study aimed to investigate changes in SARS-CoV-2 IgG antibody titers following the fourth vaccination among 28 patients undergoing hemodialysis. Blood tests were conducted at various intervals post-vaccination, with a focus on identifying factors associated with antibody levels. The IgG antibody levels rapidly increased by Day 7 post-vaccination, with a median time to peak of 11 days. Antibody titers tended to be higher in male patients than in female patients. This study sheds light on the immune response to the fourth vaccination in patients undergoing hemodialysis. As this study included a small sample size, with a short observation period, further research is warranted to comprehensively understand the effectiveness of vaccination and the benefits of additional doses of vaccine.

## 1 Introduction

Patients undergoing hemodialysis are at a higher risk of experiencing critical outcomes of the severe acute respiratory syndrome coronavirus 2 (SARS-CoV-2) infection and have higher mortality than the general population, with a reported mortality rate of 24.9% (1). Both standard and booster vaccinations reduce the risk of hospitalization and death in this population (2). Cohen-Hagai et al. (3) reported that a fourth dose of vaccine reduced rates of severe SARS-CoV-2-related hospitalization and mortality among patients undergoing hemodialysis, whereas Einbinder et al. (4) highlighted that infection risk was similar between patients vaccinated with three or four doses. Thus, the effect of a fourth dose of vaccine is unclear. Furthermore, limited data are available on IgG antibody levels in patients undergoing hemodialysis following a fourth vaccination. This study aimed to describe the trends in SARS-CoV-2 IgG antibody titers, especially the timing and factors associated with the increase after a fourth dose of vaccine in patients undergoing dialysis.

## 2 Methods

In this single-center study, we recruited 30 patients undergoing hemodialysis scheduled for their fourth dose of BNT162b2 (Pfizer-BioNTech) vaccine, who consented to participate in a series of blood tests following the vaccination. Two patients who contracted SARS-CoV-2 before receiving a fourth vaccine dose were excluded from the analysis. We determined prior infection status based on anti-nucleocapsid IgG (IgG-N) antibody levels and patient self-report. Patients received the first dose of vaccine between May 10, 2021, and July 28, 2021; the second dose between June 1, 2021, and August 18, 2021; and the third dose between January 28, 2022, and March 1, 2022. None of the patients had a history of SARS-CoV-2 infection based on regular PCR testing and self-report. In August 2022, all patients were vaccinated with a fourth dose of BNT162b2 vaccine, having previously received three doses of BNT162b2 vaccine. Blood tests for SARS-CoV-2 S1-IgG antibodies were conducted on Days 0, 2, 4, 7, 9, and 11, post-vaccination. All serological assays were performed using a chemiluminescence immunoassay (CLIA) with iFlash 3000 (YHLO Biotech, Shenzhen, China) and iFlash-2019-nCoV series (YHLO Biotech) as reagents, approved by the U.S. Food and Drug Administration. AU/mL × 1.0 was used to convert to binding antibody units (BAU/mL). The cutoff values of IgG-N antibody levels were set at 10 AU/mL according to the manufacturer’s guidelines. We assumed that the initial rise of the antibody titers after the fourth vaccination would occur during this period considering that the initial rise after the third vaccination occurred at Day 7 (3). We collected data from medical records on patients’ sex, age, medication history, comorbidities, and adverse reactions to injections. We analyzed the temporal progression of SARS-CoV-2 S1-IgG antibody levels following the fourth vaccination. Additionally, we investigated patient factors associated with S1-IgG antibody levels on the day when the highest antibody titer was observed. A linear regression model was used to examine the relationship between patient factors and antibody titers. All statistical analyses were conducted using JMP version 17, with p < 0.05 denoting statistical significance.

The study was approved by the ethics committees of Fukushima Medical University (number 2021-116) and the study was performed in accordance with the guidelines of the Declaration of Helsinki. All patients provided written informed consent before receiving the fourth dose of vaccine.

## 3 Results

The final study cohort consisted of 28 patients. The patient characteristics are shown in **Supplementary Table 1**. The mean age was 66 (interquartile range [IQR], 61–71) years. The most common cause of chronic kidney failure was diabetes (14/28, 50%), followed by unknown (6/28, 21%) and chronic glomerulonephritis (3/28, 11%). The median time since starting hemodialysis was 51 (IQR, 24–142) months. Of the 28 patients, 22 (79%) were male and 12 (44%) were aged >70 years. The patients had a high prevalence of comorbid conditions, including 27 (96%) with hypertension, 11 (39%) with diabetes, and 18 (64%) with cardiovascular disease. The mean Kt/V was 1.50 (IQR, 1.37–1.72).

**Figure 1:**
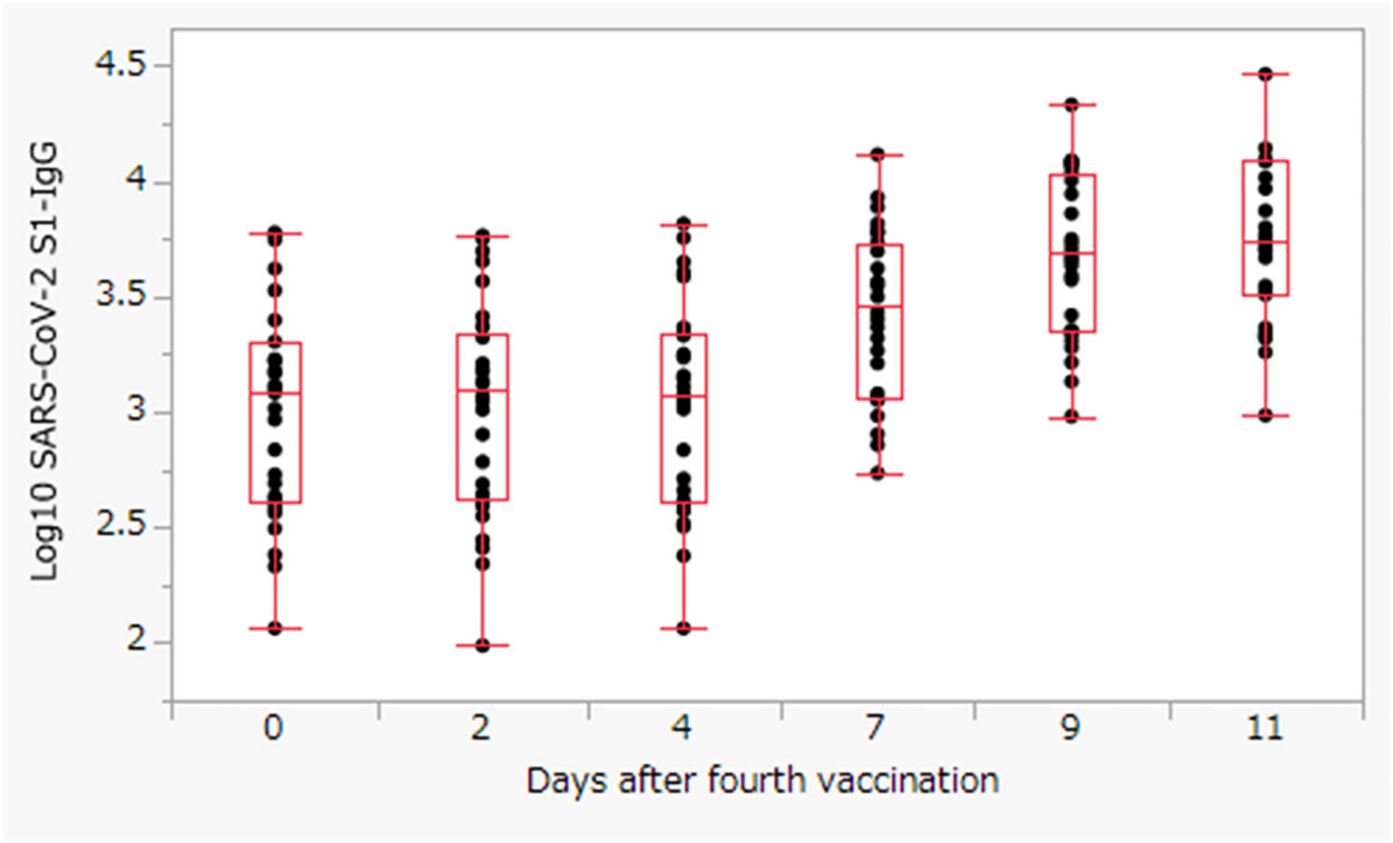
The S1-IgG levels titer after the fourth dose of BNT162b2 vaccine. The box represents the interquartile range, with the center line showing the median on the y-axis.

The S1-IgG levels increased rapidly by Day 7, and the median S1-IgG levels reached a maximum of 5479.9 (IQR: 3267.2–12199.2) AU/mL on Day 11 (**Figure 1**). Therefore, we investigated patient factors associated with S1-IgG antibody levels on Day 11 after the fourth dose of vaccine. In the univariate linear regression analysis (**Supplementary Table 2**), male patients had a higher maximum antibody titer than female patients (coefficient: 3089, 95% confidence interval [CI]: 507 to 5671, p = 0.0209). Moreover, the antibodies titers were higher in patients with low Kt/V (coefficient: 6071, 95% CI: −2189 to 14332p = 0.1429), patients with higher serum hemoglobin levels (coefficient: −1339, 95% CI: −3108 to 429, p = 0.1315), patients aged >70 years (coefficient: 1258, 95% CI: −1063 to 3580, p = 0.2754), and patients taking antihistamines (coefficient: −1494, 95% CI, −3794 to 604, p = 0.1930). However, none of these factors were statistically significant.

## 4 Discussion

This study demonstrated a rapid increase in the IgG antibody response to SARS-CoV-2 7 days after the fourth dose of vaccine in patients undergoing hemodialysis under outpatient maintenance. This finding is similar to that of Kawashima et al. (5) reporting a rapid increase in antibody levels after the third dose, which is observed 7 days post-vaccination. Furthermore, in the general population, immune protection is enhanced 7 days after the third dose of vaccine (6), which is consistent with the observed trend of increased antibody levels in our cohort. Although patients undergoing hemodialysis may have a limited increased in antibodies after the fourth dose, the results of this study suggest that the increase in antibody levels occurred within a period similar to that in the general population. Furthermore, Joo et al. (7) reported that a fourth dose of vaccine may be beneficial to protect patients undergoing hemodialysis who have not previously been infected with SARS-CoV-2 against severe infection. However, Kanai et al. (8) reported that the humoral immune response becomes blunted after the third or fourth vaccination in patients undergoing hemodialysis. Therefore, further studies are needed to determine the clinical effectiveness of booster doses of vaccine.

This study has several limitations. First, the small sample size, single-center design, and brief observation period of the study may limit the generalizability of the findings. Second, this study did not evaluate cellular immunity, which plays a crucial role in preventing severe disease. SARS-CoV-2 vaccines also affect cellular immunity, and evaluating IgG alone is insufficient. Third, this study only included patients undergoing hemodialysis and did not compare them with healthy controls. Fourth, this study lacks data on the clinical effectiveness of vaccination and did not evaluate whether the increase in antibody titers provided protection from infection. Fifth, only early post-vaccination data were available and we did not investigate the duration for antibody levels to decline after peaking. Despite these limitations, this study makes a meaningful contribution because only few studies have examined antibody changes after the fourth vaccination in patients undergoing hemodialysis. Future studies should investigate the relationship between vaccination and cellular immunity in a larger group with a longer follow-up period.

In conclusion, this study found a rapid increase in antibody titers after the fourth vaccination. Although a fourth dose of vaccine and subsequent vaccinations are potentially beneficial, a comprehensive understanding of the immune response to vaccinations requires long-term follow-up studies and additional cases.

## Data Availability

The original contributions presented in the study are included in the article/**Supplementary Material**. Further inquiries can be directed to the corresponding author.
