# Supplementary material for: SARS-CoV-2 spike protein antibody titers after the fourth dose of BNT162b2 vaccine among Japanese patients undergoing hemodialysis: a single-center study
Source: Front Immunol. 2024 Aug 21;15:1412918. doi: 10.3389/fimmu.2024.1412918 (PMC11375752; doi:10.3389/fimmu.2024.1412918)
Supplement: Supplementary file 1 [file Table1.docx]

Supplementary Material

**Supplementary Table 1. Patient characteristics (N = 28)**

| Characteristic | Value |
| --- | --- |
| Sex, male, n (%) | 22 (79%) |
| Age, years (IQR) | 66 (61–71) |
| Time since starting hemodialysis, months (IQR) | 51 (24–142) |
| Kt/V (IQR) | 1.50 (1.37–1.72) |
| Dialysis hours per week, hours, (IQR) | 12 (12-12) |
| Biochemical parameters |  |
| Serum hemoglobin levels, g/dL (IQR) | 11.6 (10.8–12.4) |
| Serum phosphorus levels, mg/dL (IQR) | 5.1 (3.8–5.9) |
| Serum albumin levels, g/dL (IQR) | 3.5 (3.2–3.6) |
| Cause of chronic kidney disease, n (%) |  |
| Diabetes | 14 (50%) |
| Unknown | 6 (21%) |
| Chronic glomerulonephritis | 3 (11%) |
| Others | 5 (18%) |
| Medications, n (%) |  |
| Steroids | 2 (7%) |
| Antihistamines | 12 (43%) |
| Anticancer drugs | 1 (4%) |
| Antiplatelet or anticoagulant drugs | 20 (69%) |
| Comorbidities, n (%) |  |
| Hypertension | 27 (96%) |
| Diabetes | 16 (57%) |
| Cardiovascular disease | 18 (64%) |
| Respiratory disease | 4 (14%) |
| Rheumatism | 1 (4%) |
| Adverse reaction, n (%) |  |
| Fever | 5 (18%) |
| Pain | 5 (18%) |
| Headache | 1 (4%) |
| Other complications | 2 (7%) |

IQR, interquartile range

**Supplementary Table 2. Univariate analysis of factors associated with SARS-CoV-2 S1-IgG antibody levels on Day 11 after the fourth dose of BNT162b2 vaccine**

| Variable | Coefficient (95% CI) | p-value |
| --- | --- | --- |
| Male | 3089 (507 to 5671) | 0.0209 |
| Age (≥ 70 years) | 1258 (−1063 to 3580) | 0.2754 |
| Kt/V | 6071 (−2189 to 14332) | 0.1429 |
| Dialysis hours per week, hours, (IQR) | 740 (−2414 to 3896) | 0.6334 |
| Biochemical parameters |  |  |
| Serum hemoglobin levels | −1339 (−3108 to 429) | 0.1315 |
| Serum phosphorus levels | 429 (−950 to 1808) | 0.5285 |
| Serum albumin levels | −3878 (−13476 to 5721) | 0.4139 |
| Medications |  |  |
| Steroids | 1035 (−3512 to 5583) | 0.6437 |
| Antihistamine | −1494 (−3794 to 804) | 0.1930 |
| Anticancer drug | 2220 (−4054 to 8495) | 0.4734 |
| Antiplatelet or anticoagulant drugs | 59 (−2459 to 2577) | 0.9619 |
| Comorbidities |  |  |
| Hypertension | 2566 (−3687 to 8819) | 0.4067 |
| Diabetes | −326 (−2731 to 2078) | 0.7823 |
| Cardiovascular disease | 1127 (−1285 to 3539) | 0.3457 |
| Respiratory disease | −837 (−4181 to 2507) | 0.6113 |
| Rheumatism | −2437 (−8699 to 3824) | 0.4309 |
| Adverse reaction |  |  |
| Fever | −85 (−3452 to 2682) | 0.7984 |
| Pain | 242 (−2827 to 3312) | 0.8723 |
| Headache | −1482 (−7793 to 4827) | 0.6331 |
| Other complications | −652 (−5212 to 3906) | 0.7708 |

CI, confidence interval
